# Periorbital Cutaneous Larva Migrans

**DOI:** 10.4269/ajtmh.26-0270

**Published:** 2026-09-22

**Authors:** Carol Lobo, Sowmya Kaimal

**Affiliations:** Department of Dermatology, St. John’s Medical College, Bangalore, India

A 43-year-old woman presented with a 1-month history of an asymptomatic, serpiginous, erythematous, raised tract on her right eyelid (Figure 1). The patient reported swimming in a freshwater body in Himachal Pradesh 1 month before lesion onset.

**Figure 1.**
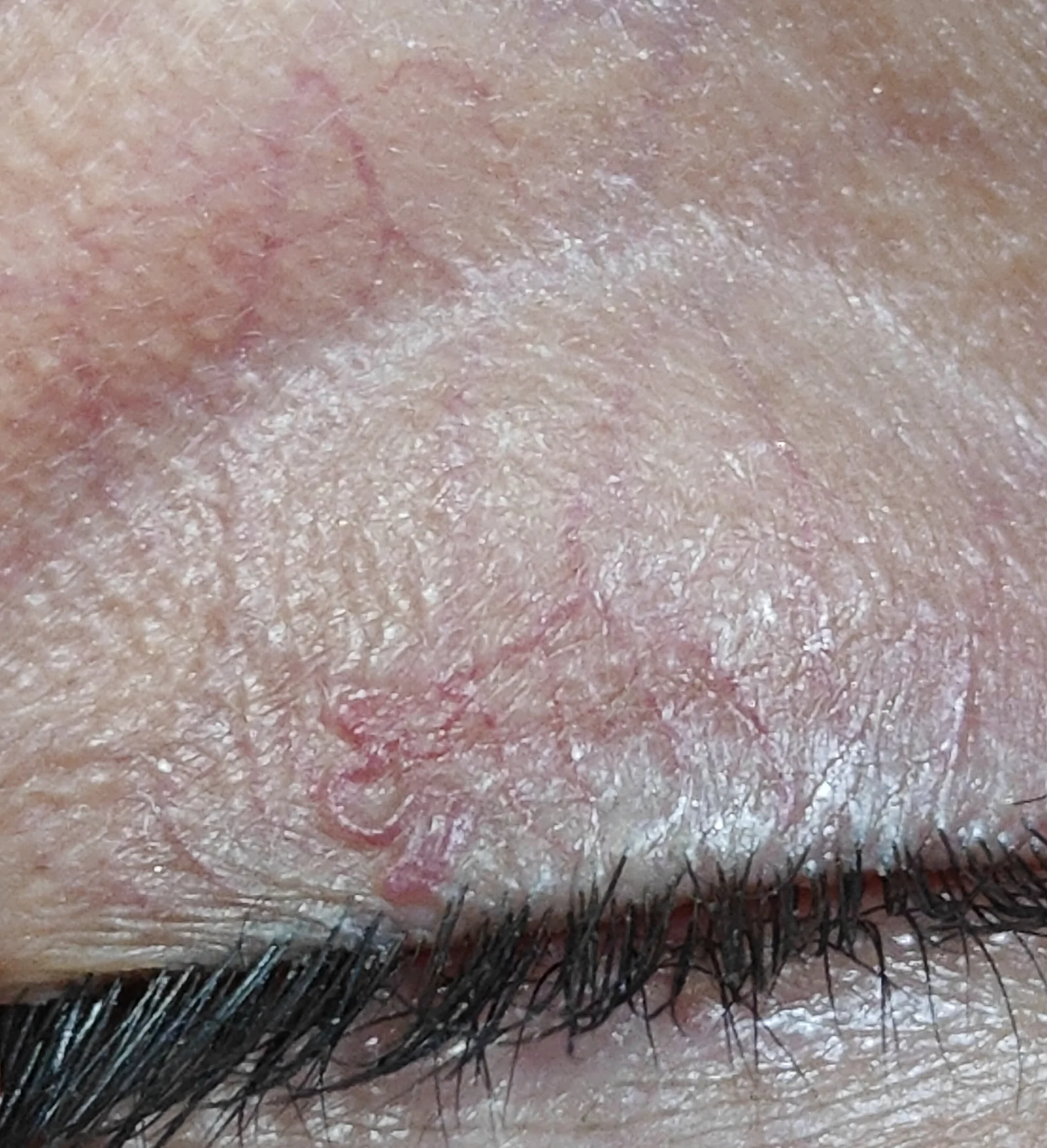
Serpiginous erythematous linear tract involving the right upper eyelid.

The diagnosis of cutaneous larva migrans was made clinically based on the characteristic serpiginous migratory tract. Cutaneous larva migrans is caused by the migration of hookworm larvae, commonly *Ancylostoma braziliense* or *Ancylostoma caninum*, within the epidermis. Infection is typically acquired through skin contact with soil or sand contaminated by animal feces.^1^ Although the source of infection could not be established in our patient, incidental exposure to contaminated soil around the freshwater body is a possible explanation.

Cutaneous larva migrans classically presents as a serpiginous erythematous tract that advances 1 to 2 cm daily; it typically involves the feet and is usually pruritic. Periorbital involvement is rare and may pose a diagnostic challenge, delaying clinical recognition.^2^

Differentials include larva currens, gnathostomiasis, and cutaneous myiasis. The characteristic morphology and subsequent therapeutic response supported the diagnosis in our patient.

The patient was treated with albendazole (400 mg daily for 3 days), resulting in marked reduction of size of the lesion within 10 days. Although cutaneous larva migrans is often self-limiting, systemic antihelminthic therapy hastens lesion resolution and limits further larval migration.^3^

## References

[1] Heukelbach J Feldmeier H, 2008. Cutaneous larva migrans. Lancet Infect Dis 8(5): 302–309. 10.1016/S1473-3099(08)70098-7 18471775

[2] Blackwell V Vega-Lopez F, 2001. Cutaneous larva migrans: Clinical features and management. Am J Clin Dermatol 2(3): 145–149. 10.1046/j.1365-2133.2001.04406.x 11531833

[3] Caumes E, 2000. Treatment of cutaneous larva migrans. Clin Infect Dis. 30(5): 811–814. 10.1086/313787 10816151

